# Relative Validity of the Eat and Track (EaT) Smartphone App for Collection of Dietary Intake Data in 18-to-30-Year Olds

**DOI:** 10.3390/nu11030621

**Published:** 2019-03-14

**Authors:** Lyndal Wellard-Cole, Juliana Chen, Alyse Davies, Adele Wong, Sharon Huynh, Anna Rangan, Margaret Allman-Farinelli

**Affiliations:** Nutrition and Dietetics Group, School of Life and Environmental Science, Charles Perkins Centre, The University of Sydney, NSW 2006, Australia; jche6526@uni.sydney.edu.au (J.C.); adav5418@uni.sydney.edu.au (A.D.); adele.wlp@gmail.com (A.W.); shuy9672@uni.sydney.edu.au (S.H.); anna.rangan@sydney.edu.au (A.R.); margaret.allman-farinelli@sydney.edu.au (M.A.-F.)

**Keywords:** diet assessment, relative validity, smartphone, young adults, apps

## Abstract

(1) Background: Smartphone dietary assessment apps can be acceptable and valid data collection methods but have predominantly been validated in highly educated women, and none specifically measured eating-out habits in young adults. (2) Methods: Participants recorded their food and beverage consumption for three days using the Eat and Track (EaT) app, and intakes were compared with three dietitian-administered 24-h recall interviews matched to the same days as the reference method. Wilcoxon signed-rank or *t*-tests, correlation coefficients and Bland–Altman plots assessed agreement between the two methods for energy and percentage energy from nutrients (%E). (3) Results: One hundred and eighty nine of 216 participants (54% females, 60% resided in higher socioeconomic areas, 49% university-educated) completed the study. There were significant differences in median energy intake between methods (*p* < 0.001), but the EaT app had acceptable agreement for most nutrient densities at the group level. Correlation coefficients ranged from r = 0.56 (%E fat) to 0.82 (%E sugars), and between 85% and 94% of participants were cross-classified into the same or adjacent quartiles. Bland–Altman plots showed wide limits of agreement but no obvious biases for nutrient densities except carbohydrate in males. (4) Conclusions: The EaT app can be used to assess group nutrient densities in a general population of 18-to-30-year olds.

## 1. Introduction

Young adults (aged 18 to 30 years) have experienced the fastest rate of weight gain of any birth cohort in Australia [1]. One factor that appears to influence the diets of people in this age group is the amount of foods eaten prepared away from home, such as fast foods. More frequent consumption of fast foods has been associated with less healthy eating habits [2]. Young adult Australians consume fast foods more frequently than other age groups [3], and spend the highest proportion of their household income on eating out [4].

There have been no recent surveys on the amount and types of foods prepared and eaten away from home by young adults in Australia. The Measuring Young adults’ Meals (MYMeals) study aims to fill this research gap [5]. Central to determining what young people are eating are valid and feasible dietary intake data collection methods.

Smartphone dietary intake methods can be acceptable and valid ways of collecting dietary data [6]. Considering that 95% of 18-to-34-year olds in Australia own a smartphone [7], it is an accessible way to collect dietary data from this age group, including those living in rural and remote locations [6,8].

Smartphone applications (apps) can be used as alternatives to traditional pen and paper or telephone food records or recalls [6]. Advantages of using electronic methods to collect dietary intake data are that entries can be completed more quickly than traditional methods [8], nutritional analysis can be conducted in real time, and researcher burden can be significantly reduced [9].

A number of commercial diet collection apps are available, however, validation studies show that these do not have good agreement with established dietary methods for some nutrients, including energy, protein, total fat, sugars, fibre and sodium [10,11]. Such differences may be due to the underlying nutrition composition databases, inadequacy of food listings available and no accounting for food preparation methods. In addition, many of the commercial apps were developed with American audiences in mind and therefore contain foods that are different to those available in Australia [10]. This makes it difficult for Australians to find and log the correct foods, which may reduce the accuracy of the nutritional data captured [12]. Further, these apps are mostly designed for weight management, and provide continuous feedback on the amounts of energy and/or nutrients consumed that may change behaviour, reducing their validity in research settings [6].

There have been three smartphone dietary recording apps (My Meal Mate, electronic Dietary Intake Assessment (eDIA) and Easy Diet Diary) that have been validated for the research setting using 24-h recalls as the reference method [13,14,15,16]. An additional app, electronic Carnet Alimentaire (e-CA, or “food record” in French), has also been evaluated favourably in a small study of 50 participants [17]. Two of these studies were conducted mostly in women in older age groups [16,17]. One was conducted in young adults, but the participants were almost exclusively university educated and of high socioeconomic status [14].

The Eat and Track (EaT) smartphone application is a new app for collection of dietary intake data, purpose-designed by the research team [18]. The aim of this study was to assess the relative validity of the EaT app with dietitian-administered 24-h recalls, examining energy and nutrient densities in a sample more inclusive of the Australian young adult population with respect to education and socioeconomic status.

## 2. Materials and Methods

### 2.1. Sample

Potential participants completed a screening and demographics questionnaire with questions on age group, educational attainment and residential postcode, to allow the socioeconomic status to be determined using Socio-Economic Indexes for Areas [19]. Participants were recruited from the overall MYMeals study population [5]. To satisfy ethics, participants had to give separate consent to opt into the validation study. This subgroup of participants was randomly allocated to complete the validation until 20% of the entire MYMeals sample was included. Potential participants were recruited across New South Wales (NSW), Australia’s most populous state. They were eligible to participate in both the MYMeals study and the present validation study if they were aged 18 to 30 years, owned a working smartphone, were English-speaking, and consumed at least one meal, snack or drink purchased outside the home per week. Participants were excluded if they did not meet the aforementioned criteria, had ever been diagnosed with an eating disorder, were not able to complete the three days required for the study or were pregnant and/or breastfeeding. Potential participants completed a screening questionnaire through the online research management platform, REDCap [20], and provided consent. The study was approved by The University of Sydney Human Research Ethics Committee (project 2016/546).

### 2.2. Eat and Track Smartphone Application (EaT App)

The EaT app was developed by nutrition and information technology experts at the University of Sydney for the purposes of data collection for the MYMeals study specifically, and is based on the e-DIA app the researchers developed previously that was validated for nutrients and food groups [14,15]. Key usability modifications to the e-DIA app were the addition of a large, branded fast food database and improved usability functions (for more information on the development of the EaT app, see [18]). The nutrition database underpinning the EaT app included 4046 foods and beverages from the Australian Bureau of Statistics’ AUSNUT 2011–2013 database [21], and 2229 food items from the largest chain outlets in Australia [18,22]. The fast food items are categorised by outlet name and the range of portion sizes of foods and beverages available at the outlet, for example, small, medium or large fries, to enhance recording and overcome previously reported difficulties in portion size estimation [14,15,17]. The fast food nutritional composition data is currently restricted to energy, protein, total and saturated fats, carbohydrates, sugars, and sodium, and does not contain micronutrients [23].

Participants were provided with written and video instructions on how to use the EaT app prior to starting the study, and could access these resources throughout the study period from the MYMeals study website [5]. Participants using the EaT app selected an eating occasion (Breakfast; Lunch; Dinner; or Snacks and Drinks) from the landing screen of the EaT app [18]. A free-text box appeared, and participants typed in the food they had eaten. Shortlists of foods appeared, and participants could scroll through the provided options, or use keyword prompts to find the food they consumed. Once the food was selected, participants chose the amount and unit of food (e.g., gram, millilitre, slice, cup, etc.), and where the food was sourced. Participants also received a portion measures booklet [24] to assist with estimating portion sizes during recording [18]. If a participant was unable to find a particular food they consumed, they could enter it manually. When entering a food manually, the app prompted participants to enter the food or individual ingredients, amounts and units consumed.

### 2.3. Procedures

After obtaining consent, participants were emailed links to download the EaT app from either the Apple App Store or Google Play, and the instructional videos on how to use the app to log their dietary intake. Participants were required to record all foods and beverages they consumed for three consecutive days. The researchers instructed participants on the days they must record their intakes. The starting days were staggered across the population to facilitate an even spread of days over the week. Participants received daily email and/or SMS prompts to remind them to log their intakes during the study period.

The participants also completed three 24-h recall telephone interviews with research dietitians. To allow all foods to be captured by both methods, the 24-h recalls were conducted the following day, but captured data for the same days that the app was used. The automated, online ASA-24 Australia [9,25] was used to conduct the recalls so that the interview process was standardised. This computerised method involved the dietitian recording all foods and drinks consumed throughout the day into the ASA-24 Australia as they interviewed the participants. Multiple passes prompt for additional information on food form, preparation methods, portion size and omitted items. The three 24-h recalls were conducted on the days following each of the data collection days. The ASA-24 Australia uses the AUSNUT 2011–2013 database [9,26], but differs from the EaT app with respect to the number of fast food items available [18].

At the conclusion of the three 24-h recalls, participants completed an online demographics questionnaire that included questions on self-reported height and weight data [5], to enable body mass index (BMI) to be calculated. Participants received a $100AUD gift voucher as compensation for their time after they had completed all study requirements.

### 2.4. Data Cleaning

All EaT app entries were checked by research dietitians in the week following the data collection days, and participants were contacted to clarify the additional manually entered food items, any obvious errors such as incorrect unit sizes, and skipped meals. However, to give a true indication of the relative validity of the EaT app, minimal changes to the data were made. Manually entered foods (*n* = 33) were matched to the nearest entry from the EaT app by one research dietitian, then checked by two others. If the participant stated brand names for entered items that were not in the original database, the Nutrition Information Panel data for that item was added to the database by the research dietitian (2% of total entries). Two Accredited Practising Dietitians each checked all the data independently for any discrepancies. These were identified and clarified until agreement was reached. All entries for the ASA-24 Australia recall were downloaded and checked.

### 2.5. Data Analysis

Daily totals for energy and each nutrient were summed, then means were calculated for each participant for the three study days. Group means and medians for energy and nutrient densities (percentage energy (%E) from protein, total and saturated fat, total carbohydrate and sugars, and sodium per 1000 kJ) were determined for both the EaT app and 24-h recall data [27]. Paired *t*-tests were conducted on normally distributed data and the Wilcoxon signed-rank test was conducted on non-parametric data to compare the three days of data from each method.

Correlations between the EaT app and 24-h recalls were assessed using Pearson product-moment correlation or Spearman rank correlation coefficients for skewed data. Quartiles of intake from each method were calculated for energy and each nutrient density. Cross-classification was calculated by the proportion of participants classified into the same, adjacent or extreme quartiles of energy or nutrient density intake by both methods.

Bland–Altman plots [28] were constructed to assess the agreement between the EaT app and 24-h recalls for the mean energy and nutrient densities.

Participants’ basal metabolic rate (BMR) was calculated using the Schofield equation [29], based on the participants’ self-reported weight, age and gender from the demographics questionnaire. Under- and over-reporters were identified using Goldberg’s cut-offs [30]. Any participants who reported consuming an average energy intake over the three days of less than 1.0× BMR were considered as under-reporters, and if they reported more than 2.4× BMR they were deemed over-reporters [31]. Twenty-eight participants (14.8%) were classified as under-reporters and six participants were classified as over-reporters (3.2%) by the reference 24-h recall data. The full sample was used for analysis, as removing mis-reporters did not significantly change results.

IBM SPSS Statistics, version 24 was used to conduct all statistical analyses, and *p*-values < 0.05 were considered statistically significant.

## 3. Results

In total, 216 participants were recruited into the validation study. Of these, five withdrew from the study for personal or employment reasons and 20 did not complete all three days of data collection, while two were deemed to fail selection criteria, leaving a final sample size of 189 participants. The mean BMI of participants was 24.9 (SD 5.0). The characteristics of the included participants are shown in Table 1. It should be noted there are slightly fewer males than the Australian population proportion of 49%, and the proportion with post-school qualifications (65%) is more than the 56% reported by the Australian Bureau of Statistics [32].

### 3.1. Comparing Intakes between 24-h Recalls and EaT App

Significantly more energy was recorded using the 24-h recalls than the EaT app for the total sample (*p* < 0.001), females (*p* < 0.01) and males (*p* < 0.001) (Table 2). However, there were no significant differences in %E from protein, total or saturated fat, or sodium densities. The EaT app recorded significantly more %E carbohydrate than the recall for the total sample (*p* = 0.03) and for males (*p* = 0.01).

### 3.2. Correlation Coefficients and Cross-Classification

Table 3 shows the correlation coefficients between the 24-h recalls and EaT app. All correlation coefficients were positive and statistically significant (*p* < 0.001). Correlations ranged from 0.56 (%E total fat) to 0.82 (%E sugars) for the total sample. Quartile cross-classification of energy and nutrient densities with 24-h recalls and the EaT app placed 84% (%E fat) to 96% (%E sugars) of participants into the same or adjacent quartile. The proportion of participants classified into the extreme quartile ranged from 0% for %E carbohydrate to 4% for %E fat.

### 3.3. Bland–Altman Plots for 24-h Recalls and EaT App

**Figure 1:**
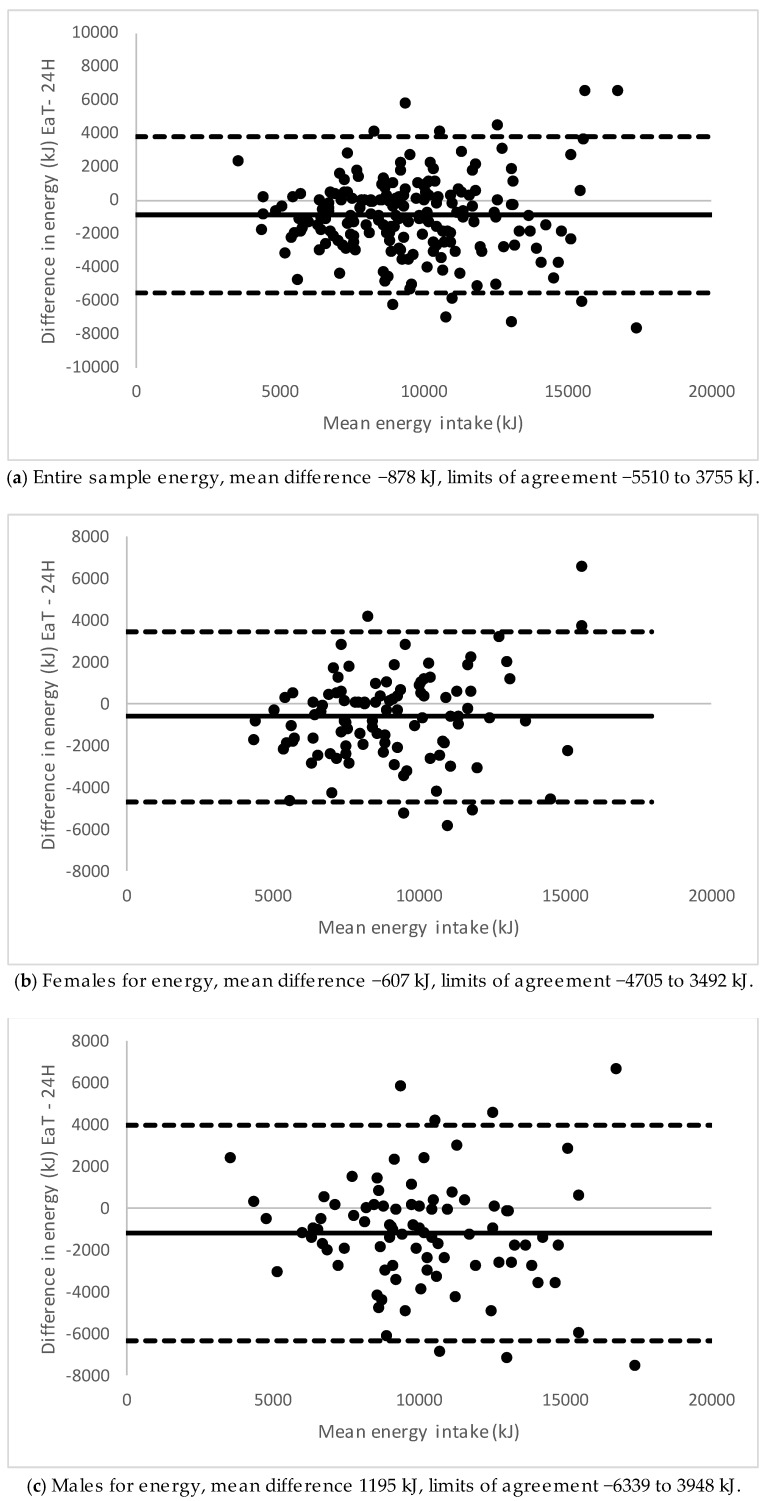
Bland–Altman plot of 24-h recalls (24H) and Eat and Track (EaT) app for energy intake. (**a**) Entire sample, (**b**) females and (**c**) males.

**Figure 2:**
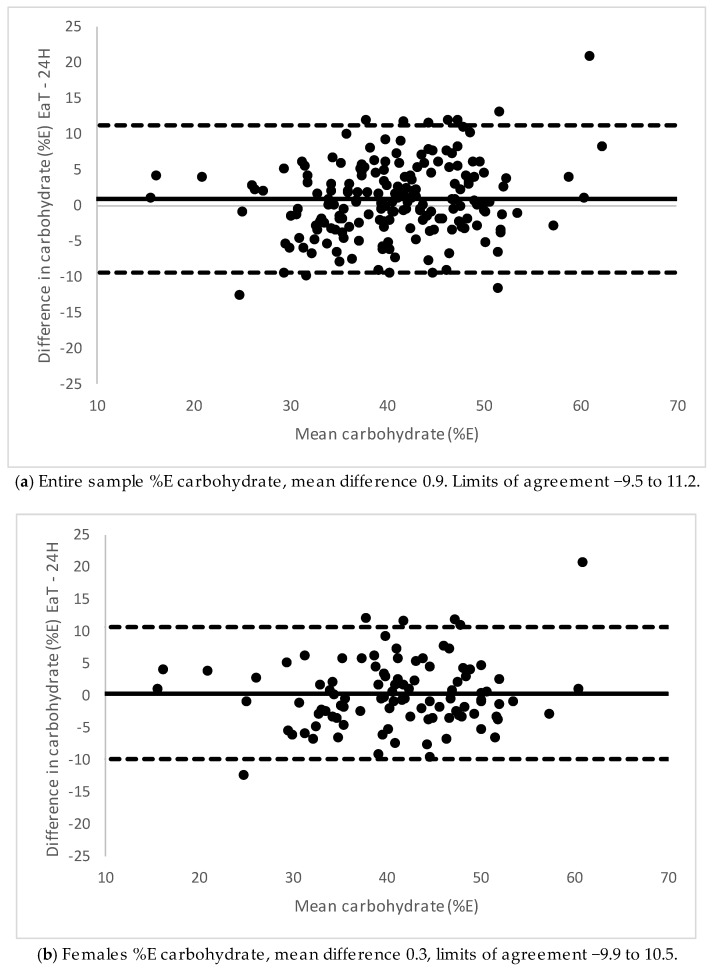

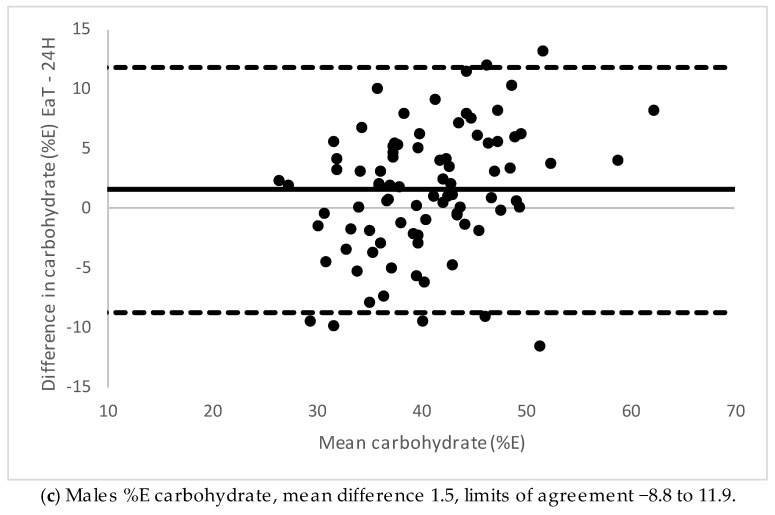
Bland–Altman plot of 24-h recalls (24H) and Eat and Track (EaT) app for %E carbohydrate. (**a**) Entire sample, (**b**) females and (**c**) males.

Bland–Altman plots showing the agreement between EaT app and 24-h recalls for energy for the total sample, males and females are presented in Figure 1. Males had a higher mean difference than females. Agreement between 24-h recalls and the EaT app for the nutrient density for carbohydrate are shown in Figure 2 because these were the nutrient densities for which a difference was found between medians. For males, carbohydrate showed underestimation at lower intakes and overestimation at higher intakes with the app compared with 24-h recalls. There were no biases detected for the other nutrient densities (plots not shown). The mean difference and 95% limits of agreement between the EaT app and 24-h recalls for energy and all nutrient densities can be seen in Table 4.

## 4. Discussion

The present study showed generally good agreement between the EaT app and 24-h recalls for nutrient densities. This finding is based on nonsignificant differences in group intakes with the exception of carbohydrates, acceptable correlation coefficients and cross-classification results. Further, the lack of bias in the Bland–Altman plots, except for carbohydrate in males, suggests that the EaT app is suitable for measuring intakes at the group level. Though there was poor agreement for energy intake, it is well established that self-reported energy intake is not a good measure of true energy intake [33]. However, energy adjustment can be used to improve estimation of nutrients [33], as has been applied in our study.

Similar to the EaT app, the apps that have been the subject of validation studies have shown good correlation with 24-h recalls, though with wide limits of agreement on Bland–Altman tests [13,14,16]. Another study in young adults validated a smartphone app that included text description, and spoken and photographic descriptions of the foods eaten using the objective measure of energy expenditure using the Sensewear armband [34]. The study reported high correlations between the methods [34]. However, it needs to be noted that of 90 participants, 13 either failed to record food intakes or wear the armband for a sufficient period of time and 21 (27%) participants were removed from the analysis because of energy misreporting [34]. As in other validation studies, the sample was mostly young educated women [34].

Due to the issues with reporting of energy intake [33], 24-h recalls are not a true ‘gold standard’ reference method of dietary intake collection. This study found low levels of underreporting via the dietitian-administered 24-h recall. In the latest Australian Health Survey, the rate of underreporting was 19% of males and 23% of females [35], higher than the rate found in our study (14.8% overall). To better assess the true validity of the EaT app, future studies using biomarkers or doubly labelled water should be conducted [33].

A strength of our study is that our sample included higher proportions of males than previous studies, various education levels and participants from both higher and lower socioeconomic status areas. This ‘real-world’ approach shows that the EaT app is likely to be useful in a diversity of population groups and may also be developed further into an app for members of the public to record and monitor their intakes.

An advantage of the EaT app in measuring diet with a focus on eating out is that participants are able to choose from a greater number of fast food options than was possible with the 24-h recall, thus increasing their likelihood of selecting the correct item. Using actual portion sizes from the fast food chains should enable better recording of these foods. For other foods, participants received a portions booklet used in national nutrition surveys to estimate serving sizes, but moving forward, inclusion of images within the app may be advantageous. There are some inherent limitations. Due to the ever-changing food supply, databases may only be accurate at one time point and quickly become outdated [36]. Fast food chains frequently offer new menu items to encourage customers into their outlets [37]. In addition, some of the differences between the EaT app and 24-h recalls may be explained by the differences in the databases used for the 24-h recalls with the ASA-24 Australia containing mostly generic fast food options [18]. Not only were there many more fast foods to choose from in the EaT app, but the fast foods in the EaT app had greater nutrient ranges and higher maximum values [18,22].

Prospective dietary data collection methods, such as the EaT app, do not rely on participants’ memories, which may be advantageous [6]. However, the selection of this method also introduces a limitation [14]. Requiring participants to record their intake in the EaT app in real time may improve the accuracy of the following 24-h recalls. However, the EaT app clears its history at 3:00 am each day [18], so participants were not able to access their data from the day before.

Overall, EaT is a promising method of collecting dietary intake data of young adults, with a particular focus on eating out. The EaT app could be used to collect data investigating the types and contributions of nutrients from different types of food outlets, and investigate effects of environmental interventions in fast food chain outlets.

## 5. Conclusions

The Eat and Track smartphone application is a valid way of collecting group nutrient density intake data in 18-to-30-year olds, with a specific focus on the nutrients of interest when frequently eating out, that is, sugars, saturated fat and sodium. To further assess the validity of the app, additional methods that do not rely on food and beverage capture and nutrient databases, such as biomarker or doubly labelled water studies, should be conducted.

## Figures and Tables

**Table 1:** Sample characteristics.

Participant Characteristics		*N* (%) ^a^
Gender	Female	102 (54)
	Male	87 (46)
Age bracket	18–24 years	105 (56)
	25–30 years	84 (44)
Body mass index	Underweight (≤18.49 kg/m^2^)	4 (2)
	Healthy weight (18.5–24.9 kg/m^2^)	116 (61)
	Overweight (25–29.9 kg/m^2^)	47 (25)
	Obese (≥30 kg/m^2^)	22 (12)
Highest education attained	Primary school or less	2 (1)
	Secondary school	64 (34)
	Trade or diploma qualification	31 (16)
	University degree	92 (49)
Socioeconomic status ^a^	Higher	114 (60)
	Lower	75 (40)

^a^ From Socio-Economic Indexes for Areas [19] based on residential postcode, lowest five deciles = lower, highest five deciles = higher.

**Table 2:** Differences in energy and nutrient density intakes recorded by the 24-h recalls and Eat and Track (EaT) app.

Energy and Nutrient Densities	Median 24-h Recall (IQR) ^c^	Median EaT App (IQR)	*p* ^d^
Entire Sample *n* = 189			
Total energy, kJ ^a^	9611 (7947–11,764)	8813 (7051–10,828)	<0.001 *
Protein, % energy ^b^	18.3 (15.2–21.6)	18.0 (15.1–21.7)	0.14
Total fat, % energy ^a^	35.8 (32.0–40.5)	35.6 (31.4–40.5)	0.47
Saturated fat, % energy ^a^	12.8 (10.6–15.5)	12.3 (10.5–15.1)	0.21
Carbohydrate, % energy ^a^	40.4 (35.3–45.7)	41.8 (35.0–47.4)	0.03 *
Sugars, % energy ^b^	15.4 (11.7–21.4)	16.4 (11.8–19.2)	0.81
Sodium, mg/1000 kJ ^b^	294.3 (239.5–349.3)	294.5 (237.2–362.3)	0.89
**Females *n* = 102**			
Total energy, kJ ^a^	9001 (7752–11,122)	8209 (6818–10,399)	<0.01 *
Protein, % energy ^a^	17.5 (14.9–20.3)	17.6 (14.8–21.0)	0.14
Total fat, % energy ^a^	36.2 (32.0–41.1)	36.6 (32.0–40.8)	0.97
Saturated fat, % energy ^a^	12.9 (10.6–16.0)	12.4 (10.6–15.6)	0.39
Carbohydrate, % energy ^a^	41.3 (35.6–47.1)	42.2 (34.6–47.6)	0.57
Sugars, % energy ^a^	18.1 (12.9–22.4)	17.2 (12.2–21.0)	0.14
Sodium, mg/1000 kJ ^a^	282.8 (229.0–354.8)	282.0 (225.3–363.9)	0.56
**Males *n* = 87**			
Total energy, kJ ^a^	10479 (8424–12985)	9140 (7359–11740)	<0.001 *
Protein, % energy ^a^	19.0 (15.5–22.7)	19.2 (15.4–21.9)	0.92
Total fat, % energy ^a^	34.9 (32.0–40.0)	34.9 (30.6–39.8)	0.29
Saturated fat, % energy ^a^	12.7 (10.5–14.7)	12.3 (9.8–14.6)	0.36
Carbohydrate, % energy ^a^	40.1 (35.1–43.7)	40.6 (35.9–47.2)	0.01 *
Sugars, % energy ^b^	14.2 (11.0–18.1)	15.0 (11.6–18.5)	0.13
Sodium, mg/1000 kJ ^b^	297.1 (249.1–349.0)	301.1 (245.0–362.1)	0.91

^a^*t*-tests for normally distributed data. ^b^ Wilcoxon signed-rank test for non-parametric data. ^c^ IQR = interquartile range. ^d^ *p* ≤ 0.05 considered significant, * denotes significant results.

**Table 3:** Correlation coefficients and cross-classification of energy and nutrient densities between the 24-h recall and Eat and Track (EaT) app.

Energy and Nutrient Densities	Correlation Coefficients ^c^	Cross-Classification into Quartiles (%)		
		Same	Same or Adjacent	Extreme
Entire Sample *n* = 189				
Total energy, kJ ^a^	0.67	50.3	90.5	2.1
Protein, % energy ^b^	0.73	53.4	93.7	2.1
Total fat, % energy ^a^	0.56	46.0	84.1	4.2
Saturated fat, % energy ^a^	0.59	49.2	84.7	3.7
Carbohydrate, % energy ^a^	0.79	52.4	95.2	0
Sugars, % energy ^b^	0.82	59.8	95.8	1.1
Sodium, mg/1000 kJ ^b^	0.56	43.3	84.7	3.2
**Females *n* = 102**				
Total energy, kJ ^a^	0.69	46.1	90.2	2.0
Protein, % energy ^a^	0.71	52.9	93.1	1.0
Total fat, % energy ^a^	0.61	48.0	86.3	2.9
Saturated fat, % energy ^a^	0.62	56.9	86.3	2.9
Carbohydrate, % energy ^a^	0.83	55.9	95.1	0
Sugars, % energy ^a^	0.82	53.9	88.2	0
Sodium, mg/1000 kJ ^a^	0.51	42.2	84.3	2.9
**Males *n* = 87**				
Total energy, kJ ^a^	0.64	54.0	85.1	2.3
Protein, % energy ^a^	0.72	56.3	90.8	2.3
Total fat, % energy ^a^	0.50	36.8	80.5	4.6
Saturated fat, % energy ^a^	0.53	43.7	85.1	4.6
Carbohydrate, % energy ^a^	0.75	50.6	93.1	1.1
Sugars, % energy ^b^	0.74	58.6	90.8	2.3
Sodium, mg/1000 kJ ^b^	0.56	40.2	85.1	4.6

^a^ Pearson’s correlation coefficients. ^b^ Spearman’s rank correlation. ^c^ All correlations were significant (*p* < 0.01).

**Table 4:** Agreement between the means of the three days of recording with Eat and Track (EaT) app with the 24-h recalls.

Nutrient	EaT Mean (SD)	24-h Recall Mean (SD)	Mean Difference (SD)	95% LOA ^a^
Total energy, kJ	9071 (2908)	9949 (2916)	−878 (2363)	(−5510, 3755)
Protein, % energy	18.8 (5.0)	18.5 (4.5)	0.3 (3.6)	(−6.8, 7.4)
Total fat, % energy	36.0 (7.0)	36.3 (6.8)	−0.3 (6.5)	(−13.0, 12.3)
Saturated fat, % energy	12.7 (3.4)	13.0 (3.4)	−0.3 (3.1)	(−6.3, 5.7)
Carbohydrate, % energy	41.3 (8.6)	40.5 (7.6)	0.9 (5.3)	(−9.5, 11.2)
Sugars, % energy	16.5 (6.5)	16.7 (6.4)	−0.2 (4.1)	(−8.2, 7.9)
Sodium, mg/1000 kJ	299.9 (89.4)	303.3 (102.5)	−3.4 (97.5)	(−194.5, 187.7)

^a^ LOA, limits of agreement.
